# Age-stratified outcomes after radical prostatectomy in a randomized setting (LAP-01): do younger patients have more to lose?

**DOI:** 10.1007/s00345-022-03945-0

**Published:** 2022-02-06

**Authors:** Sigrun Holze, Max Bräunlich, Meinhard Mende, Vinodh-Kumar-Adithyaa Arthanareeswaran, Petra Neuhaus, Michael C. Truss, Hoang Minh Do, Anja Dietel, Toni Franz, Dogu Teber, Ann-Kathrin Heilsberg, Markus Hohenfellner, Robert Rabenalt, Peter Albers, Jens-Uwe Stolzenburg

**Affiliations:** 1 Department of Urology, University of Leipzig, Liebigstraße 20, 04103 Leipzig, Germany; 2 University of Leipzig, Clinical Trial Centre Leipzig, Härtelstraße 16–18, 04107 Leipzig, Germany; 3 Institute for Medical Informatics, Statistics and Epidemiology, University of Leipzig, Härtelstraße 16–18, 04107 Leipzig, Germany; 4 Department of Urology, Klinikum Dortmund, Beurhausstr. 40, 44137 Dortmund, Germany; 5 Department of Urology, Sana Hospital Borna, Rudolf-Virchow-Straße 2, 04552 Borna, Germany; 6 Department of Urology, University of Heidelberg, Im Neuenheimer Feld 672, 69120 Heidelberg, Germany; 7 Department of Urology, Staedtisches Klinikum Karlsruhe, Moltkestraße 90, 76133 Karlsruhe, Germany; 8 Department of Urology, University of Duesseldorf, Moorenstr. 5, 40225 Düsseldorf, Germany; 9 Department of Urology, Marien Hospital Duesseldorf, Rochusstraße 2, 40479 Düsseldorf, Germany

**Keywords:** Prostate cancer, Prostatectomy, Robotic-assisted prostatectomy, Laparoscopic, Prostatectomy, Age, Continence, Potency, Quality of life

## Abstract

**Objective:**

Age is known to have an impact on outcomes after radical prostatectomy (RP). However, age differences can be investigated from a cross-sectional as well as from a longitudinal perspective. This study combines both perspectives.

**Materials and methods:**

LAP-01 is the first multicenter randomized patient blinded trial comparing outcomes after robotic-assisted and laparoscopic RP. This study stratified the entire population that received nerve-sparing surgery and was potent at baseline by the following ages: ≤ 60 years, 61–65 years, and > 65 years. Potency was assessed using the IIEF-5. The EORTC QLQ-C30 was used for global health perception and the EORTC QLQ-PR25 for urinary symptoms. Continence was assessed by the number of pads used. Longitudinal change was assessed using either validated anchor-based criteria or the 1 or 0.5-standard-deviation criterion. Worsening of continence was measured by increasing numbers of pads.

**Results:**

310 patients were included into this study. Older patients had a significantly higher risk for worsening of continence at 3 and 6 months (OR 2.21, 95% CI [1.22, 4.02], *p* = 0.009 and OR 2.00, 95% CI [1.16, 3.46], *p* = 0.013, respectively); at 12 months, the odds of worsening did not differ significantly between age groups. Potency scores were better in younger patients from a cross-sectional perspective, but longitudinal change did not differ between the age groups. In contrast, global health perception was better in older patients from a cross-sectional perspective and longitudinal decreases were significantly more common among the youngest patients, at 12 months (36.9% vs. 24.4%, *p* = 0.038).

**Conclusion:**

From a cross-sectional perspective, function scores were better in younger patients, but from a longitudinal perspective, age differences were found in continence only. In contrast, global health scores were better in older patients from a cross-sectional and longitudinal perspective.

**Trial registration:**

The LAP-01 trial was registered with the U.S. National Library of Medicine clinical trial registry (clinicaltrials.gov), NCT number: NCT03682146, and with the German Clinical Trial registry (Deutsches Register Klinischer Studien), DRKS ID number: DRKS00007138**.**

**Supplementary Information:**

The online version contains supplementary material available at 10.1007/s00345-022-03945-0.

## Introduction

Prostate cancer is the second most common malignancy in men worldwide [1], with approximately 60,000 new cases in Germany every year [2]. Radical prostatectomy (RP) is the most common type of therapy that is recommended for patients who have a life expectancy of at least 10 years [3]. Since life expectancy is increasing, RP has become a standard procedure for elderly patients, as well. However, it is known that age influences postoperative outcomes. Relevant outcomes include functional aspects such as urinary continence and potency, as well as quality of life (QOL). Many studies have shown that long-term post-RP continence rates do not differ between age groups, while post-RP erectile dysfunction (ED) is more severe among older patients [4–6]. However, these are cross-sectional comparisons that do not consider differing baseline levels. For instance, older patients have a poorer erectile function even at the baseline [7]. Wright (2008) and Brajtbord (2014) therefore investigated age differences based on longitudinal change [8, 9]. Brajtbord (2014) found that although younger patients had better overall potency scores, they were more likely to experience a severe decline. Comparisons of longitudinal changes in quality of life (QOL) showed that severe decreases of sexual bother were more prevalent in younger patients. Such findings are important for counseling prostate cancer patients prior to RP. This study strives to validate the findings described above and reports cross-sectional and longitudinal age differences based on a prospective randomized study population.

## Methods

### Study population

**Fig. 1:**
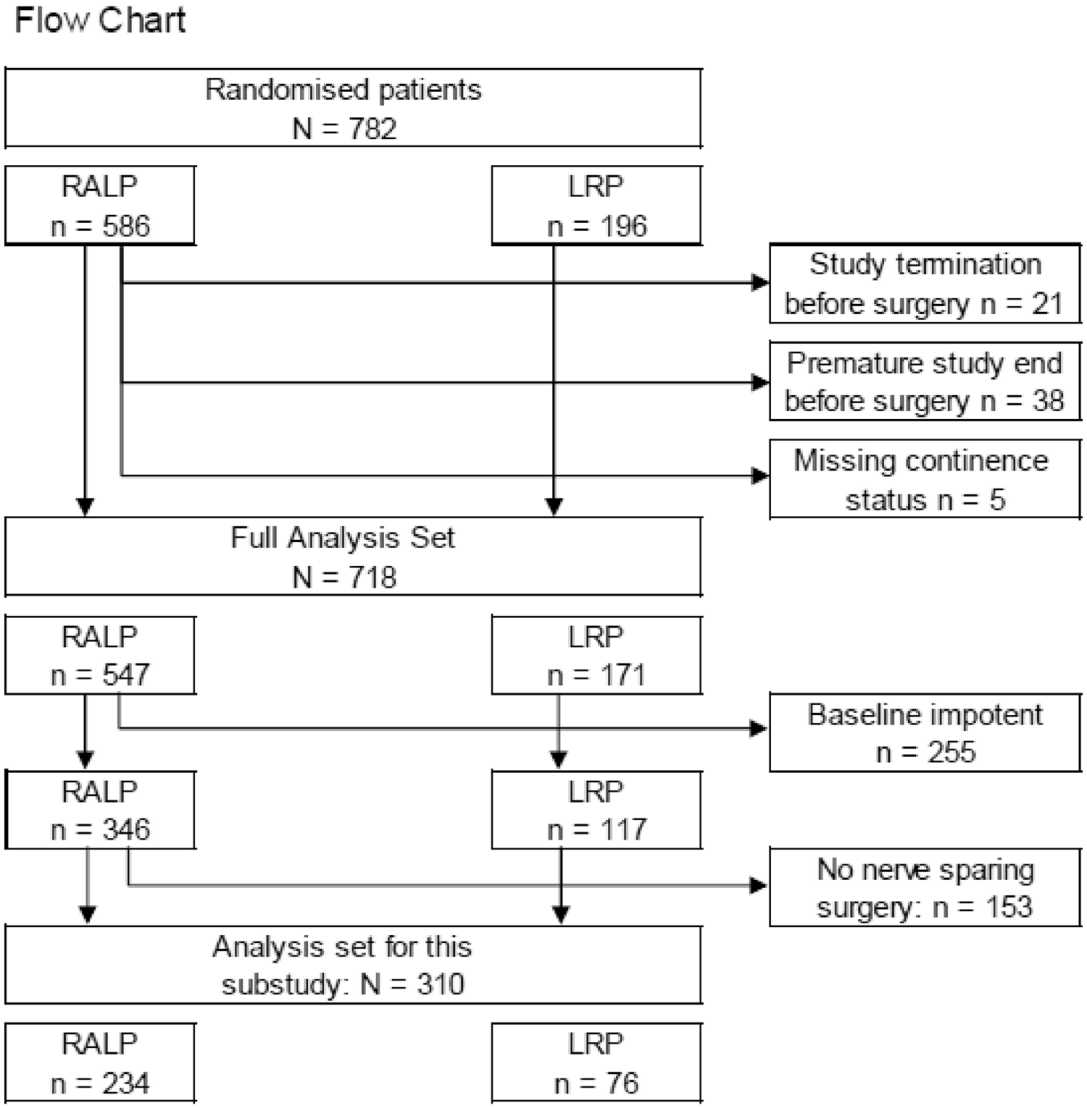
Patient flowchart

LAP-01 is a prospective randomized controlled patient blinded trial that compares patient-reported outcomes after robotic-assisted RP (RARP) and laparoscopic RP (LRP). Patients aged ≤ 75 years were recruited in four German high-volume centers between November 2014 and April 2019. 15 surgeons performed the procedures. All, except one, were experienced with over 150 procedures in each method (RARP and LRP). A detailed report on the study design and procedures has been previously published [10]. Since nerve-sparing surgery has an impact on postoperative functional outcomes [11], patients without the nerve-sparing procedure were excluded from the present study. Additionally, patients who denied having erections sufficient for intercourse at baseline were excluded. Both LAP-01 trial arms (RARP and LRP) were investigated together. The patient flowchart is shown in Fig. 1. Patients were divided into the following three age groups: ≤ 60 years, 61–65 years, and > 65 years.

### Outcome measurement

Outcomes were reported using validated questionnaires: IIEF-5 for potency, EORTC QLQ-C30 for global health perception, and EORTC QLQ-PR25 for urinary symptoms. IIEF-5 scores range from 1 to 25 points with higher scores indicating better erectile function [12]. EORTC scores range from 0 to 100 points with higher scores being favorable for the patients, except for the urinary symptoms scale, where more points indicate more problems [13, 14]. Continence outcomes were measured by the number of pads used with the following graduation: 0 pads, safety pad (without involuntary loss of urine), 1 pad, and 2 or more pads. All scores have been assessed at baseline and at 3, 6, and 12 month follow-up.

### Statistical analysis

Two different methods were used for reporting age differences. First, scores were compared pairwise in a cross-sectional manner, using the following effect measures: Cohen’s d for continuous variables, odds ratios for binary characteristics, and Delaney’s A, the probability of stochastic superiority [15] for ordinal data.

Second, changes in scores from pre- to post-RP were considered for every age group at 3, 6, and 12 months. These longitudinal changes were tested for clinical relevance using the most precise instrument available. These were the anchor-based criteria by Cocks 2012 for the EORTC QLQ-C30 [16] and the 0.5-standard-deviation (SD) criterion for EORTC QLQ-PR25 [17]. For the IIEF-5, the conventional 1 SD criterion was used, because there are no data available on sensitivity to change. Worsening of continence was defined as any change from 0 pads and the change from a safety pad to 1 pad or more. The rates of clinically relevant worsening were then compared between all age groups using tests for trend in proportions. Odds ratios are given as effect measures.

Additionally, multiple logistic regression models were built to investigate characteristics associated with long-term worsening at 12 months. The full model included age group, operation method, nerve-sparing, diabetes mellitus, obesity, use of antihypertensive drugs, placement of Rocco stitch, and the twofold interactions of operation method with age groups and nerve-sparing. The model was then simplified by stepwise backward variable elimination using the Akaike information criterion. Only the main results are reported in the results section. For a comprehensive overview, refer to the Supplementary Table 1.

Data preparation and descriptive statistics were performed by IBM SPSS Statistics, version 26. Multivariable analyses were done by R [18], including the following packages: *QoLR* [19], *dplyr, xlsx, ggplot2, and orddom*.

## Results

310 patients were included into this study. Demographic and baseline clinical characteristics are shown in Table 1. Response rates were above 90% for all scales and time points.

**Table 1:** Baseline characteristics

Demographics	Age ≤ 60 *N* = 130	Age 61–65 *N* = 88	Age > 65 *N* = 92	Effect measure* Cohen’s d/A^#^/odds ratio
	Mean ± SD	Mean ± SD	Mean ± SD	
Age at surgery [y]	55 ± 4	63 ± 1	70 ± 3	–
Body size [cm]	178 ± 6	178 ± 6	176 ± 6	*d* = 0.36
Body weight [kg]	87 ± 12	86 ± 11	83 ± 12	*d* = 0.29
BMI [kg/m^2^]	27.4 ± 3.3	27.2 ± 2.8	27.0 ± 3.1	*d* = 0.13
Karnofsky INDEX	99 ± 6	99 ± 3	99 ± 3	*d* = − 0.04

Clinical data	Median [IQR] Number (%)	Median [IQR] Number (%)	Median [IQR] Number (%)	
PSA pre-op [ng/ml]	6.61 [5.06, 9.41]	7.52 [5.52, 9.72]	7.39 [5.44, 10.13]	*d* = − 0.22
Gleason sum pre-op				*A* = 0.43
6	39 (30.0)	14 (15.9)	18 (19.8)	
7	85 (65.4)	70 (79.5)	64 (70.3)	
8	5 (3.8)	3 (3.4)	6 (6.6)	
9	1 (0.8)	1 (1.1)	3 (3.3)	
pT stage				*A* = 0.44
pT2a	5 (3.8)	7 (8.0)	7 (7.7)	
pT2b	1 (0.8)	1 (1.1)	1 (1.1)	
pT2c	103 (79.2)	65 (74.7)	56 (61.5)	
pT3a	14 (10.8)	13 (14.9)	18 (19.8)	
pT3b	6 (4.6)	1 (1.1)	8 (8.8)	
pT4	1 (0.8)	0	1 (1.1)	
Operation method				
RARP	101 (77.7)	66 (75.0)	64 (69.6)	OR 1.52
LRP	29 (22.3)	22 (25.0)	28 (30.4)	
Nerve sparing				
Unilateral	17 (13.1)	12 (13.6)	21 (22.8)	OR 0.51
Bilateral	113 (86.9)	76 (86.4)	71 (77.2)	

^*^Comparing group I (Age ≤ 60) vs. group III (Age > 65)

^#^Delanay’s A, the probability of stochastic superiority [15]

### Pad use

Except for 6 men, all patients did not use any pads at baseline. During the follow-up period, younger patients used less pads than older patients, but differences diminished at 12 months. At 3 months, the 0-pad-continence rate was 41.7% in the youngest age group as compared to 23.3% in the oldest age group. In all 3 age groups, 24.4% used a safety pad without having an involuntary loss of urine. 12 months after RP, 69.1% of the youngest patients did not use any pads compared to 60.0% in the oldest age group. The rate of patients using a safety pad ranged from 15.4% in the youngest age group to 18.9% in the oldest age group. The numbers of patients who experienced a decrease from baseline are shown in Table 2a. At 3 and 6 months, the proportion of the oldest patients who experienced a decrease was significantly higher than in the youngest age group. This finding was also reflected by the odds of worsening that were twice as high for the oldest as compared to the youngest age group at 3 and 6 months (see Table 2a). However, at 12 months, the rates of worsening did not differ significantly. In the logistic regression model, the odds of worsening were more than three times smaller for patients who underwent bilateral nerve-sparing as compared to patients with unilateral nerve-sparing (OR 0.29, 95% CI [0.13, 0.61], *p* < 0.001).

**Table 2:** Numbers of patients with relevant deteriorations^†^

	Age ≤ 60 Number (%)	Age 61–65 Number (%)	Age > 65 Number (%)	Effect measure OR [95% CI]^#^	*P* value*
(a) Pad use					
3 months	74 (58.3)	56 (65.1)	68 (75.6)	2.21 [1.22, 4.02]	0.009
6 months	50 (40.0)	40 (46.5)	52 (57.1)	2.00 [1.16, 3.46]	0.013
12 months	38 (30.9)	25 (29.1)	34 (37.8)	1.36 [0.77, 2.41]	0.32
(b) Urinary symptoms					
3 months	82 (65.6)	47 (54.7)	49 (54.4)	0.64 [0.36, 1.16]	0.085
6 months	60 (49.2)	37 (43.0)	30 (33.0)	0.52 [0.28, 0.94]	0.019
12 months	50 (41.0)	27 (31.4)	20 (22.2)	0.42 [0.21, 0.80]	0.004
(c) IIEF-5					
3 months	105 (83.3)	78 (90.7)	75 (84.3)	1.07 [0.48, 2.43]	0.74
6 months	99 (79.8)	70 (81.4)	73 (82.0)	1.15 [0.54, 2.49]	0.68
12 months	93 (75.6)	64 (74.4)	69 (76.7)	1.06 [0.53, 2.13]	0.88
(d) Global health					
3 months	59 (47.2)	38 (44.2)	40 (44.4)	0.90 [0.50, 1.60]	0.67
6 months	49 (39.5)	31 (36.0)	27 (29.7)	0.65 [0.35, 1.19]	0.14
12 months	45 (36.9)	20 (23.3)	22 (24.4)	0.56 [0.29, 1.05]	0.038

^**†**^Relevant deteriorations as defined in the methods section

^#^Odds ratio comparing group III (Age > 65) vs. group I (Age ≤ 60)

*Test for trend in proportions

### Urinary symptoms

Urinary symptom scores were better in younger patients from baseline to 12 months. There were moderate cross-sectional age differences at baseline, but these diminished during the follow-up. At 12 months, cross-sectional age differences were negligible. As can be seen in Table 3a, younger patients had larger decreases at 3 and 6 months. At 12 months, the oldest patients had better scores than at baseline, while younger patients had persistent decreases. This finding was underlined by the rates of worsening (see Table 2b). At 6 and 12 months, a significantly higher proportion of the youngest patients had relevant decreases as compared to the older patients. The logistic regression model confirmed that older patients had a lower risk of worsening as compared to the youngest patients (age 61–65: OR 0.56, 95% CI [0.30, 1.04], *p* = 0.061, age > 65: OR 0.31, 95% CI [0.16, 0.60], *p* < 0.001). The odds of worsening of patients with bilateral nerve-sparing were less than half compared to patients with unilateral nerve-sparing (OR 0.46, 95% CI [0.23, 0.92], *p* = 0.025).

**Table 3:** Mean scores of the EORTC urinary symptoms scale, IIEF-5, and EORTC global health scale^†^

	Age ≤ 60 Mean ± SD	Age 61–65 Mean ± SD	Age > 65 Mean ± SD	Cohen’s d (I vs. III)
(a) Urinary symptoms*				
Baseline	11.9 ± 11.8	12.7 ± 10.4	19.2 ± 13.8	− 0.57
3 months	24.9 ± 16.7	23.6 ± 14.1	28.9 ± 17.4	− 0.23
6 months	18.6 ± 13.7	17.3 ± 11.6	21.5 ± 15.7	− 0.20
12 months	16.9 ± 14.5	14.3 ± 12.0	17.3 ± 13.1	− 0.03
(b) IIEF-5				
Baseline	20.2 ± 4.5	19.4 ± 5.0	18.2 ± 5.2	0.43
3 months	7.9 ± 6.1	6.5 ± 5.0	5.2 ± 5.5	0.46
6 months	8.9 ± 6.6	7.9 ± 6.2	5.7 ± 5.7	0.52
12 months	10.2 ± 6.8	9.4 ± 7.1	6.2 ± 6.5	0.60
(c) Global health				
Baseline	75.8 ± 20.0	77.8 ± 15.8	78.3 ± 15.8	− 0.13
3 months	70.8 ± 17.5	75.8 ± 15.7	74.1 ± 15.4	− 0.19
6 months	74.3 ± 16.6	77.8 ± 15.5	78.4 ± 17.4	− 0.24
12 months	74.8 ± 19.6	79.8 ± 15.9	81.2 ± 13.4	− 0.37

^**†**^EORTC scores range from 0 to 100, IIEF-5 score (German version) ranges from 1 to 25

*Higher scores indicate more problems

#### IIEF-5

IIEF-5 scores were better in younger patients from baseline to 12 months (see Table 3b). These cross-sectional age differences were of moderate size considering the effect measure Cohen’s d. However, rates of worsening did not differ significantly between age groups (see Table 2c) and decreases were generally very large. Even at 12 months, scores remained far below baseline levels. The logistic regression model showed that bilateral nerve-sparing was associated with less than half the odds of worsening as compared to unilateral nerve-sparing (OR 0.41, 95% CI [0.21, 0.81], *p* = 0.01). Older patients had a lower risk of worsening than the youngest patients. However, because of the interaction effect LRP x age group, this effect was only strong for RARP patients (see Supplementary Table 1).

### Global health perception/general QOL

Cross-sectional age differences were trivial at baseline and at 3 months. However, at 6 and 12 months, the oldest patients had slightly better scores than the youngest patients (see Table 3c). When considering the anchor-based criteria for longitudinal change by Cocks 2012 [16], almost half of the study population experienced a relevant decreases at 3 months. At 12 months, a significantly higher percentage of the youngest patients had persistent decreases as compared to the oldest patients (see Table 2d). The odds of worsening, as seen in the logistic regression model, were half the size for patients aged 61–65 (OR 0.52, 95% CI [0.27, 0.98], *p* = 0.039) and patients aged > 65 (OR 0.50, 95% CI [0.26, 0.94], *p* = 0.028) when compared to patients aged ≤ 60. Bilateral nerve-sparing was associated with less than half the odds of worsening as compared to unilateral nerve-sparing (OR 0.38, 95% CI [0.17, 0.82], *p* = 0.012). However, this effect disappeared in LRP patients (see Supplementary Table 1).

## Discussion

Our study investigated several aspects of the health of RP patients. We found that post-RP continence rates were better in younger patients from a cross-sectional as well as from a longitudinal perspective. However, differences at 12 months were not significant. In terms of potency, younger patients had better overall scores at all time points. Yet, from a longitudinal perspective, rates of worsening did not differ between age groups. On the other hand, global health perception was better in older patients from a cross-sectional and from a longitudinal perspective. The scores for urinary symptoms were worse in older patients at all time points, yet severe decreases were more common in younger patients. In summary, younger patients tended to have better functional scores, but they were more likely to experience decreases of QOL. Moreover, our data highlight the problem of only using cross-sectional data to compare post-RP outcomes between age groups. As seen in the IIEF-5, there can be cross-sectional differences between age groups that do not exist from a longitudinal perspective. In the EORTC urinary symptoms scale, cross-sectional and longitudinal age differences were inverse.

Many studies have investigated age differences in continence outcomes after RP. Most of them found that long-term continence rates did not differ between age groups, while recovery of continence was faster in younger patients [4, 8, 9, 20]. Our data confirm these findings. However, mean continence rates remained below baseline levels. These continence rates are lower than reported elsewhere, which may be due to several reasons. First, many of the previous studies might have been subject to selection bias because of their retrospective design [5, 6, 8, 9]. Moreover, some of them were conducted by only a single surgeon [4, 5, 20]. These factors may have led to better continence outcomes which may not be representative of the everyday situation in German hospitals. We therefore assume that our data better reflect the real situation of prostate cancer patients in Germany than previous studies. Considering that almost all patients did not use any pads at baseline, we believe that incontinence as an adverse effect of RP should not be underestimated when counseling patients.

In terms of potency, previous authors stated that potency rates in younger patients were superior to those in older patients [4–6, 8, 9, 20]. This finding was equally confirmed by our data. However, it is important to note that potency scores were already better in younger patients at the baseline. We therefore assume that the cross-sectional superiority of younger patients solely reflects their better baseline scores but not a better tolerance of the surgical procedure. This hypothesis is emphasized by the fact that the rates of worsening did not differ between age groups. Moreover, attention must be paid to the size of the decreases: at 12 months, there was a mean decrease of more than 2 standard deviations (SD) from baseline; 75.6% of all patients experienced a decrease of at least 1 SD. These values are also worse than reported elsewhere, which is probably due to the same reasons as in the continence domain.

There are not many age-stratified studies that investigated function and QOL at the same time. Two of them were published by Wright (2008) and Brajtbord (2014) [8, 9]. Both applied the UCLA-PCI scales urinary bother and sexual bother which indicate how patients perceive their respective functional states. While Wright found that decreases of urinary bother were more common among older patients, Brajtbord could not find any age differences in this domain. We did not apply the UCLA-PCI but the EORTC QLQ. The urinary symptom scale refers to the frequency of urination; pain during urination; and the impact of urination on night sleep, leaving the home, and daily activities. It is therefore more robust than the UCLA-PCI scale urinary bother which only consists of one single question. As opposed to Brajtbord and Wright, in our study, population worsening of urinary symptoms was more common among younger patients. A possible explanation for this finding is that when looking more profoundly into urinary problems, older patients might be used to a variety of urinary symptoms even before RP, while younger patients may develop such symptoms only following RP. This finding is especially interesting, because the urinary symptoms scores are opposed to the continence scores. The same applies to the global health scale. It refers to the patient’s global health perception and his overall quality of life. Younger patients had worse scores than older patients, even though they tended to have better function scores. In contrast, older patients had better global health scores but worse function scores. These findings suggest that the assessment of post-RP function differs according to age.

## Conclusion

Our study revealed a contrast between post-RP function and the assessment of post-RP QOL. Younger patients had a lower risk for worsening of continence (significant at 3 and 6 months but not at 12 months following RP). Despite the fact that younger patients had better scores in erectile function, worsening of the erectile function did not differ between age groups at any time point. In contrast, younger patients had a significantly higher risk for worsening of QOL at 12 months after RP. These findings suggest that the assessment of post-RP function differs according to age.

## Supplementary Information

Below is the link to the electronic supplementary material.

Supplementary file1 (DOCX 18 KB)
