# Supplementary material for: Age-stratified outcomes after radical prostatectomy in a randomized setting (LAP-01): do younger patients have more to lose?
Source: World J Urol. 2022 Feb 6;40(5):1151–8. doi: 10.1007/s00345-022-03945-0 (PMC9085667; doi:10.1007/s00345-022-03945-0)
Supplement: Supplementary file 1 — Supplementary file1 (DOCX 18 KB) [file 345_2022_3945_MOESM1_ESM.docx]

**Supp. Tab. 1 Predictors of Relevant Deteriorations**

|  | Odds Ratio | 95%-CI | P value |
| --- | --- | --- | --- |
| a) Pad-Use |  |  |  |
| Operation Method: LRP | 0.42 | [0.10, 1.71] | 0.22 |
| Interaction: LRP x Bilateral Nerve-sparing | 4.65 | [1.01, 21.4] | 0.044 |
| Nerve-sparing: Bilateral | 0.29 | [0.13, 0.61] | <0.001 |
| b) Urinary Symptoms |  |  |  |
| Age: 61-65 | 0.56 | [0.30, 1.04] | 0.061 |
| Age: >65 | 0.31 | [0.16, 0.60] | <0.001 |
| Operation Method: LRP | 1.58 | [0.88, 2.83] | 0.12 |
| Nerve-sparing: Bilateral | 0.46 | [0.23, 0.92] | 0.025 |
| c) IIEF-5 |  |  |  |
| Age: 61-65 | 0.52 | [0.25, 1.06] | 0.066 |
| Age: >65 | 0.24 | [0.10, 0.55] | 0.001 |
| Nerve-sparing: Bilateral | 0.41 | [0.21, 0.81] | 0.01 |
| Interaction: LRP x Age ≤60 | 0.99 | [0.40, 2.44] | 0.99 |
| Interaction: LRP x Age 61-65 | 1.81 | [0.63, 5.17] | 0.26 |
| Interaction: LRP x Age >65 | 3.13 | [1.08, 9.09] | 0.03 |
| d) Global Health |  |  |  |
| Age: 61-65 | 0.52 | [0.27, 0.98] | 0.039 |
| Age: >65 | 0.50 | [0.26, 0.94] | 0.028 |
| Operation Method: LRP | 0.42 | [0.09, 1.91] | 0.25 |
| Interaction: LRP x Bilateral Nerve-sparing | 3.50 | [0.68, 18.1] | 0.13 |
| Nerve-sparing: Bilateral | 0.38 | [0.17, 0.82] | 0.012 |

**Supp. Tab. 2 Histopathology and Adjuvant Therapy**

|  |  | Age ≤60  Number (%) | Age 61-65  Number (%) | Age >65  Number (%) |
| --- | --- | --- | --- | --- |
| Histopathology | | | | |
| Lymph node metastases | N0  N1  NX | 76 (58.5)  4 (3.1)  50 (38.5) | 59 (67.0)  4 (4.5)  25 (28.4) | 62 (86.9)  4 (4.4)  24 (26.7) |
| Resection status | R0  R1 | 119 (91.5)  11 (8.5) | 82 (93.2)  6 (6.8) | 80 (87.9)  11 (12.1) |
| Adjuvant Therapy | | | | |
| 1 Month | Adjuvant Therapy | 3 (2.3) | 1 (1.1) | 1 (1.1) |
|  | Radiotherapy | 2 (1.5) | 1 (1.1) | 0 |
|  | Hormone Therapy | 2 (1.5) | 0 | 1 (1.1) |
| 3 Months | Adjuvant Therapy | 5 (4.0) | 3 (3.6) | 6 (6.9) |
|  | Radiotherapy | 5 (4.0) | 3 (3.6) | 5 (5.7) |
|  | Hormone Therapy | 2 (1.6) | 1 (1.2) | 4 (4.6) |
| 6 Months | Adjuvant Therapy | 7 (5.7) | 4 (4.7) | 9 (9.9) |
|  | Radiotherapy | 7 (5.7) | 4 (4.7) | 9 (9.9) |
|  | Hormone Therapy | 0 | 1 (1.2) | 4 (4.4) |
| 12 Months | Adjuvant Therapy | 7 (5.4) | 5 (5.7) | 7 (7.6) |
|  | Radiotherapy | 7 (5.8) | 5 (6.1) | 6 (6.7) |
|  | Hormone Therapy | 0 | 2 (2.4) | 2 (2.2) |
